# Brain Aging: Uncovering Cortical Characteristics of Healthy Aging in Young Adults

**DOI:** 10.3389/fnagi.2017.00412

**Published:** 2017-12-11

**Authors:** Sahil Bajaj, Anna Alkozei, Natalie S. Dailey, William D. S. Killgore

**Affiliations:** Social, Cognitive, and Affective Neuroscience Laboratory, Department of Psychiatry, University of Arizona, Tucson, AZ, United States

**Keywords:** limbic system, healthy aging, cortical measures, functional networks, cortical thickness, cortical volume, cortical surface area

## Abstract

Despite extensive research in the field of aging neuroscience, it still remains unclear whether age related cortical changes can be detected in different functional networks of younger adults and whether these networks respond identically to healthy aging. We collected high-resolution brain anatomical data from 56 young healthy adults (mean age = 30.8 ± 8.1 years, 29 males). We performed whole brain parcellation into seven functional networks, including visual, somatomotor, dorsal attention, ventral attention, limbic, frontoparietal and default mode networks. We estimated intracranial volume (ICV) and averaged cortical thickness (CT), cortical surface area (CSA) and cortical volume (CV) over each hemisphere as well as for each network. Averaged cortical measures over each hemisphere, especially CT and CV, were significantly lower in older individuals compared to younger ones (one-way ANOVA, *p* < 0.05, *corrected for multiple comparisons*). There were negative correlations between age and averaged CT and CV over each hemisphere (*p* < 0.05, *corrected for multiple comparisons*) as well as between age and ICV (*p* = 0.05). Network level analysis showed that age was negatively correlated with CT for all functional networks (*p* < 0.05, *corrected for multiple comparisons*), apart from the limbic network. While age was unrelated to CSA, it was negatively correlated with CV across several functional networks (*p* < 0.05, *corrected for multiple comparisons*). We also showed positive associations between CV and CT and between CV and CSA for all networks (*p* < 0.05, *corrected for multiple comparisons*). We interpret the lack of association between age and CT of the limbic network as evidence that the limbic system may be particularly resistant to age-related declines during this period of life, whereas the significant age-related declines in averaged CT over each hemisphere as well as in all other six networks suggests that CT may serve as a reliable biomarker to capture the effect of normal aging. Due to the simultaneous dependence of CV on CT and CSA, CV was unable to identify such effects of normal aging consistently for the other six networks, but there were negative associations observed between age and averaged CV over each hemisphere as well as between age and ICV. Our findings suggest that the identification of early cortical changes within various functional networks during normal aging might be useful for predicting the effect of aging on the efficiency of functional performance even during early adulthood.

## Introduction

By the term aging, we usually refer to time-dependent changes in functional and structural characteristics of living organisms that could be responsible for reduction in homeostasis and functional capacity (Hung et al., 2010). A decline in some cognitive abilities, even among healthy elderly, is well documented to be associated with aging (Glisky, 2007). For many elderly persons such declines become sufficiently serious when they reach a point where it becomes difficult for the individual to live independently. The basic cognitive functions such as attention, memory and perception are few among several behavioral skills, which are most affected by age (Glisky, 2007; Mahoney et al., 2010). On a molecular level, aging is reported to be responsible for cell shrinkage and a decline in the quality of several cellular mechanisms including protein synthesis, which could further lead to the formation of improper folding of protein aggregates and set the basis for the emergence of various neurodegenerative diseases (Gaczynska et al., 2001). For example, Alzheimer’s disease is more common in individuals over the age of 65 compared to individuals in their 30, 40, or 50 s^1^ and 75% of all strokes occur in individuals over the age of 65.^2^ It is well accepted that aging has wide ranging effects on cells, molecules, and cognition, as well as on brain volume. It has also been found that after age 40, the volume of the brain reduces at the rate of around 5% per decade (Svennerholm et al., 1997). In particular, it has been shown that the volume of the frontal and temporal lobes decreases with age, especially after the age of 44 and 47 respectively (Bartzokis et al., 2001; Peters, 2006). Therefore, aging neuroscience is one of the most crucial and timely fields dedicated to understanding the underlying mechanisms behind several such age-related brain disorders.

Despite extensive progress in the field of aging neuroscience, it remains unclear how different functional networks respond to healthy aging in terms of their structural characteristics such as cortical thickness (CT), cortical surface area (CSA), and cortical volume (CV), and whether the pattern remains consistent across the whole brain and its different functional networks. It is very important to detect possible brain loss associated with early aging in order to identify the onsets of neurodegenerative diseases in advance. Second, the combined use of multiple cortical measures in the field of aging neuroscience is still in its infancy and it is not clear how the lack of detailed age-related cortical surface data might affect our understanding of normal aging; detailed age-related examination of structural characteristics could substantially advance our understanding of the fundamental relationships between aging and brain tissue loss.

Structural measures CT, CSA and CV are also known to display specific age related regional variations across the brain’s cortical surface (White et al., 2010). In a study with a broad age-range (18–93 years) of healthy subjects, wide-spread cortical thinning reflecting significant atrophy was reported in middle-aged adults (age range = 41–57 years), especially in the prefrontal as well as the temporal and parahippocampal cortex (Salat et al., 2004). Older age was associated with smaller surface area in the dorsolateral and orbitofrontal cortex (DLPFC and OFC), and with greater cortical thickness in the DLPFC and anterior cingulate cortex (Dotson et al., 2015). Global gray matter volume has also been reported to decrease significantly with age, with steeper declines in males compared to females (Good et al., 2001). Very limited age-oriented studies with a special focus on functional networks, especially involving young adults, have reported the effect of healthy aging on these networks. Using positron emission tomography, reduced functional brain activity during perceptual decision-making was found to be associated with greater age in older adults (mean age 67 years) (Grady et al., 1994). More recently, studies have investigated the effects of aging on cognitive and executive function abilities, including working memory and inhibitory control (Hertzog et al., 2008; Turner and Spreng, 2012). On the whole, these studies reported a constant maturation of the adult brain for roughly five decades, followed by degeneration. Therefore, most of the studies on human aging have concentrated on either functional brain responses in healthy older adults or on individuals already suffering from some kind of age related neurogenerative disease. In addition, most studies have been region of interest (ROI) based rather than whole brain network analyses. There is presently limited information about the effect of age on multiple cortical characteristics of different functional brain networks, especially in healthy young adults. We, therefore, examined the association between age and cortical measures in a sample of healthy young adults.

In this study, we explored the effect of age on CT, CSA, and CV averaged over each hemisphere, as well as on intracranial volume (ICV). Further, we parcellated the whole brain into seven functional networks (network 1: visual network, network 2: somatomotor network, network 3: dorsal attention network, network 4: ventral attention network, network 5: limbic network, network 6: fronto-parietal network and network 7: default mode network) (Yeo et al., 2011) for left and right hemispheres individually and calculated subject-wise brain cortical measures such as CT, CSA, and CV for each of these seven networks. Based on previous research and the goals of the present study, we hypothesized that (i) age would be negatively correlated with averaged CT, CSA, and CV over each hemisphere, intracranial volume (ICV) as well as with averaged CT, CSA and CV over each of the seven functional network and (ii) there would a significant association between CT, CSA, and CV for each of the seven functional networks. Testing these hypotheses would clarify the fundamental relationships between normal aging and changes in cortical structure of the brain.

## Materials and Methods

### Participants and Data Acquisition

**FIGURE 1:**
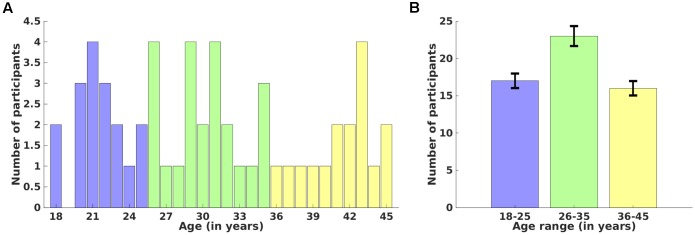
Distribution of participants over age. Age-wise (18–45 years) **(A)** and age-range (18–25, 26–35, and 36–45 years) **(B)** distributions over number of participants. Error bars represent the standard deviation of the mean value.

Fifty-six healthy adult participants between 18 and 45 years of age (mean age = 30.8 ± 8.1 years, 29 males) participated in this study. Detailed age-wise (18–45 years) (A) and age-range (Group 1: 18–25, Group 2: 26–35, and Group 3: 36–45 years) (B) distributions over number of participants are shown in **Figure 1**. Participants were screened via a comprehensive telephone interview and were excluded for any history of psychiatric, neurological, or significant medical problems (e.g., heart problems, diabetes), including head injury with loss of consciousness longer than 30 min, sleep disorders, or current use of psychotropic medications that could affect neuroimaging. Participants were also excluded for any history of drug or alcohol treatment or current use of illicit substances. Current alcohol use was required to be lower than the Center for Disease Control criteria for excessive alcohol use^3^. Written informed consent was obtained from each participant before the experiment and the study protocol was approved by the Institutional Review Boards of McLean Hospital and Partners Healthcare, as well as by the United States Army Human Research Protections Office. Other unrelated behavioral data from this sample have been reported elsewhere (Killgore et al., 2013), but the cortical thickness and cortical surface area measures reported here are novel and have not been previously reported. We recorded high-resolution anatomical magnetic resonance imaging (MRI) data using a 3-Tesla Siemens whole brain MR scanner located at the McLean Hospital Imaging Center. Each participant was instructed to rest, relax and try his/her best to stay motionless during the entire scan. Anatomical data for each participant was acquired using a 3D magnetization-prepared rapid acquisition gradient echo (MPRAGE) sequence which consisted of 128 sagittal slices (thickness = 1.33 mm, voxel resolution = 1 × 1 × 1.33 mm, field of view (FOV) = 256 mm) with TR/TE/FA/inversion time of 2100 ms/2.25ms/12°/1100 ms.

### Data analysis

We used the “recon-all” pipeline in FreeSurfer (version 6.0)^4^ installed on a Debian 8 LINUX machine to process anatomical images for all the participants. This processing involved motion-correction, brain extraction (i.e., removal of skull, skin, neck and eyes), automated transformation to Talairach co-ordinate system, intensity correction, volumetric segmentation, and whole brain parcellation into seven functional networks (visual network, somatomotor network, dorsal attention network, ventral attention network, limbic network, fronto-parietal network, and default mode network) using the Yeo atlas (Yeo et al., 2011). These seven networks and their corresponding regions/components are summarized in **Table 1**. The naming of all the regions constituting each network was performed by overlaying annotation file (split components) of 7 networks over 7-network parcellation in FreeView. The measures of mean CT, mean CV and mean CSA were evaluated individually for the left and the right hemisphere to determine any significant differences in these measures across different age groups. We also estimated the correlations between age and each of these measures averaged over each hemisphere and ICV. For network level analysis, the correlation analysis between all three cortical measures and age as well as within cortical measures was performed. All the correlation analysis was performed after extracting subject-wise CT, CV and CSA measures from all seven functional networks for each hemisphere. For multiple comparisons, the Holm–Bonferroni technique (Holm, 1979) was used to adjust the *p*-value of 0.05 across all correlations of CT, CSA, and CV with age and for all correlations within cortical measures, such as between CV and CT, CV and CSA, and CT and CSA.

**Table 1:** List of cortical regions/components within each functional network.

Networks	Anatomical locations/Components
Network 1 (Visual)	LH/RH: Visual
Network 2 (Somatomotor)	LH/RH: Somatomotor
Network 3 (Dorsal Attention)	LH/RH: Posterior (Post central cortex stretches posteriorly parietal), Frontal eye fields, Precentral ventral
Network 4 (Ventral Attention)	LH: Parietal operculum, Temporal occipital, Frontal operculum, Lateral prefrontal cortex, Medial (frontal cortex through medial parietal) RH: Temporal occipital parietal, Precentral, Frontal operculum, Lateral prefrontal, Ventral prefrontal, Medial (medial frontal cortex through medial parietal)
Network 5 (Limbic)	LH/RH: Temporal pole, Orbital frontal cortex
Network 6 (Frontoparietal)	LH: Parietal, Temporal, Dorsal prefrontal cortex, Lateral prefrontal, Orbital frontal, Ventral prefrontal, Precuneus, Cingulate, Medial posterior prefrontal RH: Parietal, Temporal, Ventral prefrontal, Lateral prefrontal, Precuneus, Cingulate, Medial posterior prefrontal
Network 7 (Default mode)	LH: Parietal, Temporal, Prefrontal, Posterior cingulate, Parahippocampal RH: Parietal, Temporal, Ventral prefrontal, Medial prefrontal, Posterior cingulate

*LH/RH, left hemisphere/right hemisphere. Please note that the above reported regions within each network are based on approximate anatomical locations and components extracted using Yeo2011_17Networks_N1000.split_components.annot in FreeView*.

## Results

We used one-way ANOVAs to estimate the differences in CT, CSA, and CV across the three age groups for each hemisphere. We also report the correlation between age and averaged CT, CSA, and CV over each hemisphere and between ICV and age. Further, Pearson’s correlation coefficients and corresponding *p*-values, corrected for multiple comparisons using Holm–Bonferroni (Holm, 1979), were calculated to examine the association between each of the cortical measures (CT, CSA, and CV) and age as well as within each pair of cortical measures (between CV and CT, CV and CSA, and CT and CSA).

### Age Related Cortical Changes across Hemispheres and within Functional Networks

#### Cortical Measures across Hemispheres and Age

We calculated the average of all three cortical measures (CT, CSA, and CV) over the left and right hemisphere individually.

##### Left hemisphere

**FIGURE 2:**
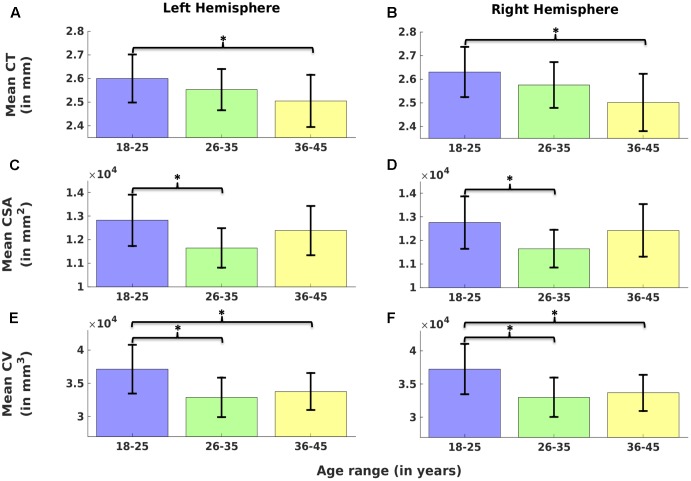
Comparison of cortical measures (Cortical thickness: CT, cortical surface area: CSA, and cortical volume: CV) across age groups. Mean CT **(A,B)**, mean CSA **(C,D)** and mean CV **(E,F)** over left **(A,C,E)** and right hemisphere **(B,D,F)** are plotted across three age groups (G1: 18–25, G2: 26–35, and G3: 36–45). Error bars represent the standard deviation. Significant differences are denoted by ^∗^(one-way ANOVA, *p <* 0.05, corrected for multiple comparisons).

**FIGURE 3:**
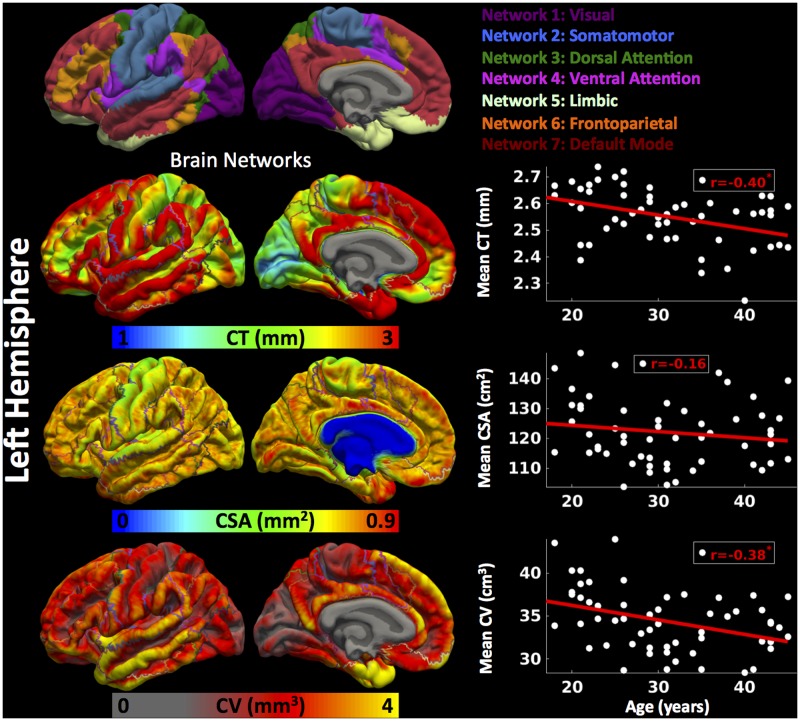
Brain parcellation and age-related changes in mean cortical measures for left hemisphere. Left hemispheric brain parcellation into seven functional networks and vertex-wise maps for cortical thickness (CT in mm), cortical surface area (CSA in mm^2^) and cortical volume (CV in mm^3^). Linear plots showing age-related significant reduction in mean CT (in mm), non-significant reduction in mean CSA (cm^2^) and significant reduction in mean CV (cm^3^) over left hemisphere.

Cortical thickness was significantly lower in group 3 versus group 1, CSA was significantly lower in group 2 versus group 1, and CV was significantly lower in group 3 versus 1 and group 2 versus 1 (**Figure 2**) (one-way ANOVA, *p* < 0.05, adjusted for multiple comparisons). For the left hemisphere (**Figure 3** and **Table 2**), we found a significant decrease in mean CT with age (*r* = -0.40, *p* < 0.05). We did not observe a significant decrease in mean CSA with age (*r* = -0.16, *p* > 0.1) but there was significant negative correlation between mean CV and age (*r* = -0.38, *p* < 0.05).

**Table 2:** Correlation coefficients between cortical measures and age.

Laterality	Mean	Networks						
		N1	N2	N3	N4	N5	N6	N7
**Correlation coefficients between**								
*CT and age*								
LH	-0.40**	-0.31*	-0.37**	-0.43*	-0.43**	-0.16	-0.32*	-0.44**
RH	-0.47**	-0.40**	-0.37**	-0.46**	-0.51**	-0.25	-0.47**	-0.48**
*CSA and age*								
LH	-0.16	-0.09	-0.11	-0.14	-0.09	-0.21	-0.15	-0.17
RH	-0.12	-0.03	-0.01	-0.04	-0.16	-0.18	-0.20	-0.16
*CV and age*								
LH	-0.38**	-0.27	-0.30	-0.34*	-0.32	-0.31	-0.40**	-0.42**
RH	-0.41**	-0.27	-0.24	-0.34*	-0.40**	-0.37**	-0.47**	-0.46**
**Partial correlation coefficients *(gender as covariate)* between**								
*CT and age*								
LH	-0.41**	-0.33*	-0.37**	-0.43**	-0.44**	-0.17	-0.32*	-0.44**
RH	-0.47**	-0.40**	-0.36**	-0.46**	-0.50**	-0.24	-0.46**	-0.48**
*CSA and age*								
LH	-0.32**	-0.18	-0.27	-0.25	-0.21	-0.32	-0.31	-0.33
RH	-0.27*	-0.13	-0.14	-0.14	-0.30	-0.26	-0.36	-0.29
*CV and age*								
LH	-0.53**	-0.38**	-0.43**	-0.43**	-0.44**	-0.40**	-0.56**	-0.57**
RH	-0.55**	-0.39**	-0.35**	-0.44**	-0.52**	-0.41**	-0.63**	-0.60**

*N1–N7, Network 1–Network 7; CT, cortical thickness; CSA, cortical surface area; CV, cortical volume; LH/RH, left hemisphere/right hemisphere. ^∗^Significant at 0.05 ≤ *p* < 0.1 *(corrected for multiple comparisons).*^∗∗^Significant at *p* < 0.05 (corrected for multiple comparisons).*

##### Right hemisphere

**FIGURE 4:**
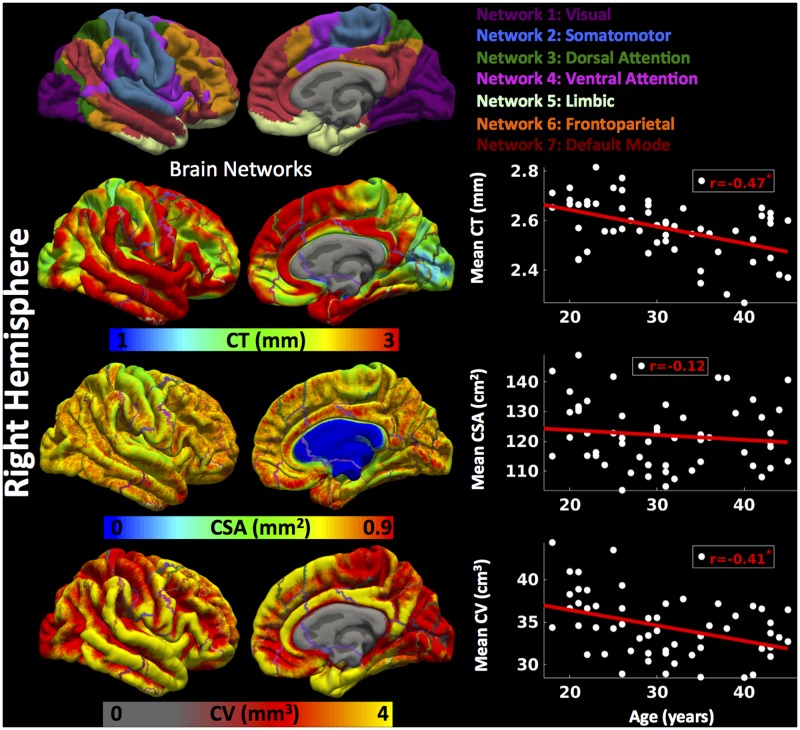
Brain parcellation and age-related changes in mean cortical measures for right hemisphere. Right hemispheric brain parcellation into seven functional networks and vertex-wise maps for cortical thickness (CT in mm), cortical surface area (CSA in mm^2^) and cortical volume (CV in mm^3^). Linear plots showing age-related significant reduction in mean CT (in mm), non-significant reduction in mean CSA (cm^2^) and significant reduction in mean CV (cm^3^) over right hemisphere.

Cortical thickness was significantly lower in group 3 versus group 1, CSA was significantly lower in group 2 versus group 1, and CV was significantly lower in group 3 versus 1 and group 2 versus 1 (**Figure 2**) (one-way ANOVA, *p* < 0.05, adjusted for multiple comparisons). For the right hemisphere (**Figure 4** and **Table 2**), correlation patterns were similar to those observed in the left hemisphere, i.e., there was a significant decrease in mean CT with increasing age (*r* = -0.47, *p* < 0.05), a non-significant reduction in mean CSA with age (*r* = -0.12, *p* > 0.1) but again a significant negative correlation between mean CV and age (*r* = -0.41, *p* < 0.05).

After regressing out the effect of ‘gender,’ the all three cortical measures (mean CT, mean CSA, and mean CV) for each hemisphere showed significant decrease with age.

**FIGURE 5:**
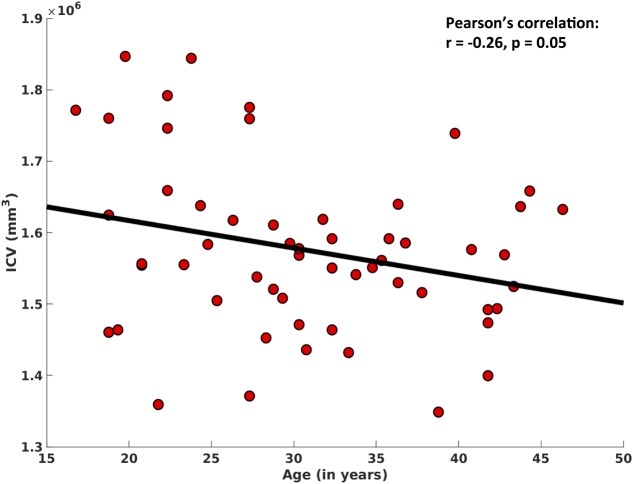
Correlation between intracranial volume (ICV) and age. After regressing out the effect of ‘gender,’ here we show a significant negative association between ICV and age.

In addition, ICV also showed significant decreases with age (*r* = -0.26, *p* = 0.05), after controlling for ‘gender’ (**Figure 5**).

#### Cortical Measures across Functional Networks and Age

We evaluated mean CT, mean CSA, and mean CV for each of the functional networks for each participant for the left and right hemisphere separately.

##### Left hemisphere

**FIGURE 6:**
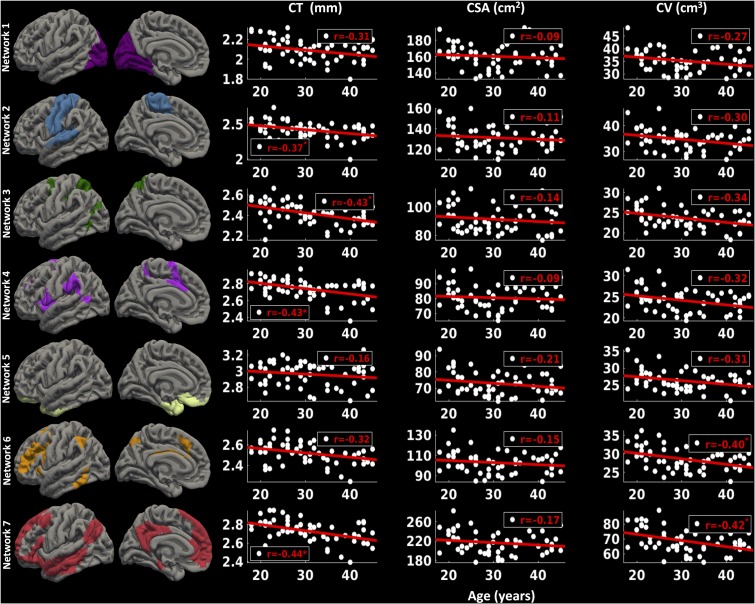
Network-wise age-related changes in cortical measures for left hemisphere. Linear plots showing significant or trend toward significant reduction in cortical thickness (CT in mm) (^∗^*p* < 0.05, corrected for multiple comparisons), non-significant reduction in cortical surface area (CSA in cm^2^) and significant or trend toward significant reduction in cortical volume (CV in cm^3^) (^∗^*p* < 0.05, corrected for multiple comparisons) for seven functional networks on left hemisphere.

For the left hemisphere (**Figure 6**), we found that there was a non-significant negative trend between mean CT and age for network 1 (*r* = -0.31, *p* < 0.1) and network 6 (*r* = -0.32, *p* < 0.1), significant negative correlations between mean CT and age for network 2 (*r* = -0.37, *p* < 0.05), network 3 (*r* = -0.43, *p* < 0.05), network 4 (*r* = -0.43, *p* < 0.05), and network 7 (*r* = -0.44, *p* < 0.05), but no significant correlation between CT and age for network 5 (limbic network) (*r* = -0.16, *p* > 0.1). We did not observe a significant relationship between CSA and age for any of the seven functional networks (*p* > 0.1). Finally, we found a significant decrease in CV in network 6 (*r* = -0.40, *p* < 0.05) and network 7 (*r* = -0.42, *p* < 0.05) with age and a trending negative correlation between CV and age for network 3 (*r* = -0.34, *p* < 0.1).

##### Right hemisphere

**FIGURE 7:**
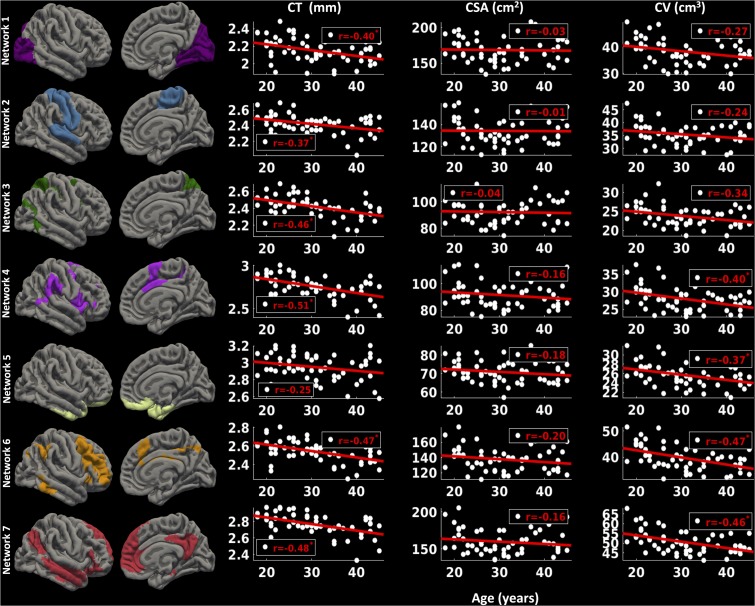
Network-wise age-related changes in cortical measures for right hemisphere. Linear plots showing significant reduction in cortical thickness (CT in mm) (^∗^*p* < 0.05, corrected for multiple comparisons), non-significant reduction in cortical surface area (CSA in cm^2^) and significant or trend toward significant reduction in cortical volume (CV in cm^3^) (^∗^*p* < 0.05, corrected for multiple comparisons) for seven functional networks on right hemisphere.

For the right hemisphere (**Figure 7**), we found a significant decrease in CT for all the functional networks (-0.37 ≤ *r* ≤-0.51, *p* < 0.05) except network 5 (limbic network) (*r* = -0.25, *p* > 0.1) with age. However, no significant relationship between CSA and age for any of the functional networks was found (*p* > 0.1). However, there was a trend for a decrease in CV with age for network 1 (*r* = -0.27, *p* < 0.1), network 2 (*r* = -0.24, *p* < 0.1), and network 3 (*r* = -0.34, *p* < 0.1), and significant decrease in CV with age for networks 4, 5, 6, and 7 (-0.37 ≤ *r* ≤-0.47, *p* < 0.05).

Consistent with above findings, after controlling for the effect of ‘gender’, we found significant negative correlations between mean CT and age for all the networks (networks 1–4 and network 6, bilaterally), except network 5 (limbic network), while there was no significant correlation between mean CSA and age for any of the networks. However, after controlling for the effect of ‘gender,’ we found significant negative correlations between mean CV and age for all the networks.

We have summarized the above findings in **Table 2**.

### Dependence of CV on CT and CSA

Further, we evaluated the association among all three (CV, CT, and CSA) cortical measures.

#### Left hemisphere

**FIGURE 8:**
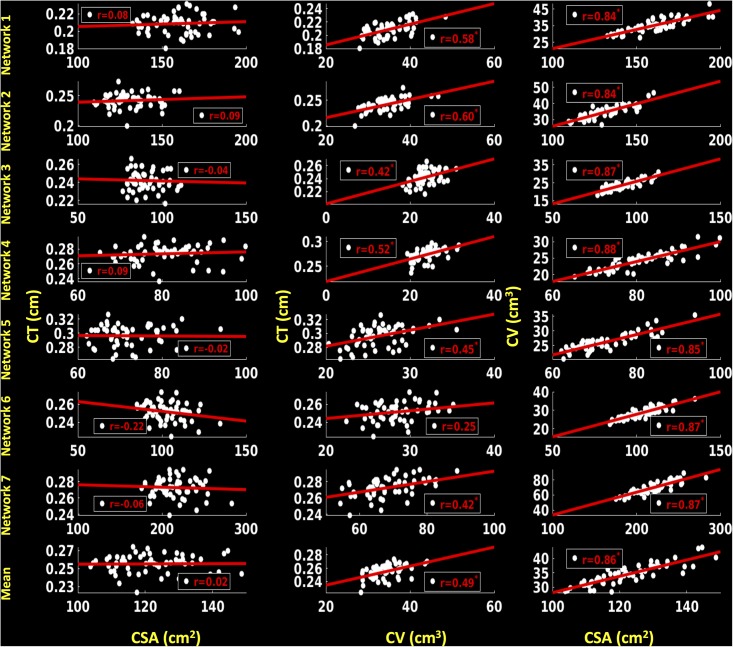
Correlations between cortical surface area (CSA) and cortical thickness (CT), cortical volume (CV) and CT and between CSA and CV for left hemisphere. Linear plots showing non-significant association between CSA (cm^2^) and CT (cm), significant or trend toward significant positive association between CV (cm^3^) and CT (cm) (^∗^*p* < 0.05, corrected for multiple comparisons), and significant positive association between CSA (cm^2^) and CV (cm^3^) (^∗^*p* < 0.05, corrected for multiple comparisons) for seven functional networks and mean over seven functional networks on left hemisphere.

For the left hemisphere (**Figure 8**), we did not find a significant association between CSA and CT for any of the functional networks (*p* > 0.1) and between mean CSA and mean CT (*r* = 0.02, *p* > 0.1). We found a non-significant positive trend (*r* = 0.25, *p* < 0.1) between CV and CT for network 6 (frontoparietal network) and significant positive correlations for the other six functional networks (0.42 ≤ *r* ≤ 0.60, *p* < 0.05). Additionally, there was a significant positive correlation between mean CV and mean CT (*r* = 0.49, *p* < 0.05). We also found significant positive associations between CSA and CV for each functional network (0.84 ≤ *r* ≤ 0.88, *p* < 0.05) as well as between mean CSA and mean CV (*r* = 0.86, *p* < 0.05).

#### Right hemisphere

**FIGURE 9:**
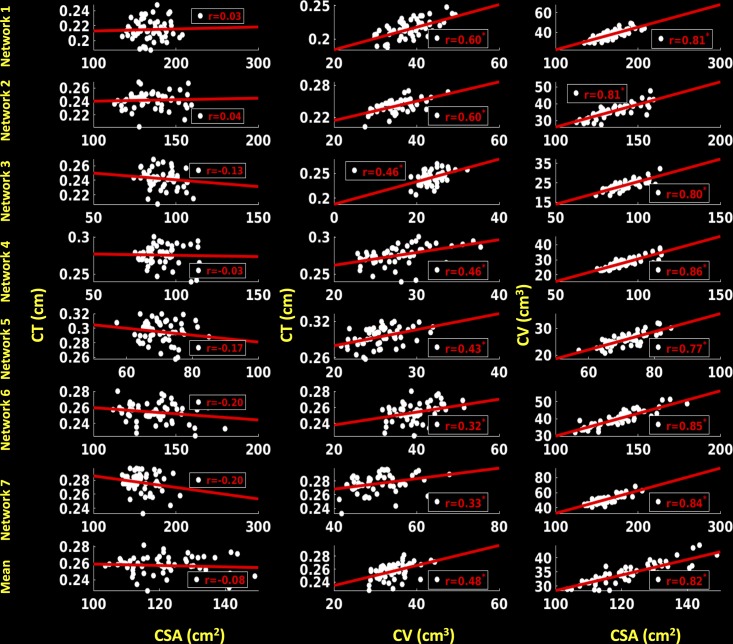
Correlations between cortical surface area (CSA) and cortical thickness (CT), cortical volume (CV) and CT and between CSA and CV for right hemisphere. Linear plots showing non-significant association between CSA (cm^2^) and CT (cm), significant or trend toward significant positive association between CV (cm^3^) and CT (cm) (^∗^*p* < 0.05, corrected for multiple comparisons), and significant positive association between CSA (cm^2^) and CV (cm^3^) (^∗^*p* < 0.05, corrected for multiple comparisons) for seven functional networks and mean over seven functional networks on right hemisphere.

For the right hemisphere (**Figure 9**), we did not find any significant associations between CSA and CT for any of the functional networks (*p* > 0.1), and no associations between mean CSA and mean CT (*p* > 0.1). We found significant positive correlations between CV and CT for all the functional networks (0.32 ≤ *r* ≤ 0.60, *p* < 0.05), as well as between mean CV and mean CT (*r* = 0.48, *p* < 0.05). We also found significant positive associations between CSA and CV for each functional network (0.77 ≤ *r* ≤ 0.86, *p* < 0.05) and between mean CSA and mean CV (*r* = 0.82, *p* < 0.05).

## Discussion

In this study of young healthy adults, we observed significant (i) differences in CT, CSA, and CV across different age groups (ii) associations between increasing age and reductions in brain averaged cortical measures such as CT, CSA, and CV, and (iii) negative association between age and ICV. At the network level, cortical measures, especially CT, within six functional networks – visual, somatomotor, dorsal attention, ventral attention, fronto-parietal and default mode was found to reduce with age. Notably, we found a significant association between age and CT in all six networks except the limbic network, suggesting that the CT of this latter network may remain stable during the early adult years. Contrary to expectations, there were no associations between age and CSA for any of the functional networks. Negative associations between age and CV were observed in most of the networks before regressing out the effect of gender and in all of the networks after regressing out the effect of gender. Significant associations were found between CV and CT, as well as between CV and CSA, whereas, CSA and CT appear to be independent. These findings indicate that (1) the limbic network is the least susceptible to age-related changes among those networks studied here in young healthy controls, and (2) CT is one of the most sensitive measures to identify age related cortical changes in young adults. Additionally, the widespread dynamics of CT, CSA, and CV across several functional networks reflect non-uniform cortical changes within the brain. These findings suggest that single modality approaches to characterizing age related changes in the cortex may be insufficient to understand the dynamics of healthy aging.

### Stability of the Limbic Network with Age

Previous research has identified age-related changes in cortical measures such as thickness, surface area and gray matter volume across different regions of interest using voxel-based and surface based measures (Salat et al., 2004; Lemaitre et al., 2012). In a study of 70 healthy men between the ages of 19 and 76 years, significant age-related loss in gray matter volume in the frontal and temporal lobes was reported (Bartzokis et al., 2001). It was suggested that a reduction in the number of large neurons and an increase in the proportion of small neurons contributed to an overall reduction in cortical volume (Terry et al., 1987; Bartzokis et al., 2001). However, network level changes in cortical characteristics have rarely been investigated in prior work. In addition, most of the previous studies reported inconsistent trend effects of aging on surface-based cortical measures, especially cortical surface area (Salat et al., 2004; Fjell et al., 2006; Rettmann et al., 2006; Dickerson et al., 2009). In this study, we found a nearly consistent global reduction in mean CT and unchanged mean CSA of each hemisphere as well as for all the functional networks with the exception of the limbic network. Consistent with a study by Grieve and colleagues (Grieve et al., 2005), we found a preserved limbic network in terms of non-significant changes in magnitude of CT and CSA with age. In that study, significant preservation, relative stability, and sparing of the medial temporal lobe including the entorhinal and parahippocampal cortices across the entire age range were reported. In addition, in younger and older adults, robust functional activation during detection of emotional faces was found in the amygdala, suggesting that the amygdala remains relatively preserved with aging (St Jacques et al., 2010; Campbell et al., 2012; Hampson et al., 2012). Significant stability of the hippocampal region until the 7th decade followed by a sharp decline after the 8th decade was also reported (Allen et al., 2005). Several other studies support the notion that regions associated with the limbic network remain relatively preserved in aging humans (Raz et al., 1997), aging rats (Rapp and Gallagher, 1996), and aging primates (Keuker et al., 2003).

Our findings did not show how the observed age-structure associations correspond to changes in actual cognitive performance. However, reduced cortical thickness is considered one of the possible mechanisms responsible for significant deviation in global cognition resulting in lower cognitive reserve (Ferreira et al., 2017), as measured by the Wechsler Adult Intelligence Scale (WAIS). To some extent, higher cognitive reserve restricts the clinical symptoms following brain pathology (Stern, 2009), and shields and compensates for the effect of cortical thinning (Ferreira et al., 2016). It is also possible that cognitive reserve mechanisms may protect against the changes in brain structure within the limbic network. The present findings reported in our study, as well as those of previous studies showing a protective role of cognitive reserve (Freret et al., 2015; Ferreira et al., 2017) and memory decline with age (Peters, 2006), suggest that the basis for age-related decline in cognitive skills is not entirely due to structural loss of a specific brain region within the limbic network. It is intriguing to speculate that the relative preservation of the limbic network with age in our sample and prior evidence of spared amygdala activation in older adults may suggest a greater reliance upon emotional encoding/retrieval strategies with increasing age.

### Instability of Six Functional Networks with Age

In this study, significant reductions in CT and CV as a function of age for six functional networks reflect a widespread pattern of age-related cortical changes. This widespread pattern of age-related structural changes was also evident from lower CT, CSA, and CV in older individuals versus younger individuals. Significant negative associations between age and averaged CT and CV over each hemisphere as well as between age and ICV were also observed. There is abundant evidence showing age-related declines in brain activation and cognitive performance but limited evidence showing age-related *structural* changes in multiple regions of the human brain (Trollor and Valenzuela, 2001; Scahill et al., 2003). Age-related functional changes, however, could be a possible direct/indirect indicator of associated age-related structural modifications. Such changes in brain morphology have been extensively investigated in postmortem as well as *in vivo* magnetic resonance imaging studies (Kemper, 1994; Raz, 2000), with most showing age related weakening of the brain structure (i.e., decline in weight, volume, and white matter integrity) and global decline of cognitive functions.

Below we discuss our findings showing instability of each of the six networks with age:

#### Visual (Network 1) and Somatomotor (Network 2) Networks

Our findings are consistent with prior work which found a prominent decrease in cortical thickness of the visual network constituting the occipital cortex and within or near primary visual cortex, especially in calcarine cortex, and the somatomotor network constituting primary somatosensory and motor cortices (pre/post central gyrus and central sulcus) (Salat et al., 2004). Raz and colleagues also reported significant (trends toward significant) age-related changes in the occipital cortex. However, these were smaller in magnitude when compared to changes within the prefrontal cortex (Raz et al., 1997). These findings are inconsistent with the ‘last in, first out’ model of memory decline. Although this model is valid during development and degradation of myelin (Kemper, 1994), future work is required to validate the same hypothesis for cortical measures.

#### Dorsal Attention (Network 3) and Ventral Attention (Network 4) Networks

We reported a significant decrease in CT, no significant change in CSA, and significant decreases in CV for dorsal attention network (DAN) and ventral attention network (VAN) with increasing age. Recently, in a study involving attention and short-term memory tasks, healthy aging was associated with preserved activity in DAN but reduced activation in the VAN as a function of short term memory (Kurth et al., 2016). The authors of that study suggested that the activation in DAN remains preserved during healthy aging but diminishes in VAN over the same period. In a large study of healthy non-demented older adults (mean age 80.4 years), Mahoney and colleagues found significant positive associations between age and alertness and performance on an executive attention task (Mahoney et al., 2010). In that study, the authors postulated that the reduction in alertness and performance could be attributed to limited attentional resources and a deteriorating functional role of the prefrontal cortex with aging (West, 1996; Raz et al., 1997).

#### Frontoparietal (Network 6) and Default Mode (Network 7) Networks

We reported trends toward significant decreases in CT, no significant changes in CSA and significant decreases in CV for frontoparietal network (FPN) with increasing age. In addition, we reported significant age-related decreases in CT, no significant changes in CSA, and significant decreases in CV for default mode network (DMN). Several functional studies have shown age related decrease in functional connectivity within the FPN and DMN. For example, during a cognitive control task, the FPN, which plays a key role in executive functions, was found to be more activated in younger adults compared to older adults (Campbell et al., 2012). Further, it was found that in young, middle-aged, and older adults, there were correlations between age and the intrinsic brain activity within the DMN (Damoiseaux et al., 2008; Hampson et al., 2012). Recently, a study involving older (mean age 64.9 years) and younger adults (mean age 20.6 years) showed that the efficiency of the DMN, fronto-parietal control network and cingulo-opercular network declined with age (Geerligs et al., 2015). In another study, aging was associated with reduced connectivity within the DMN (Vidal-Pineiro et al., 2014).

### Significance and Possible Underlying Mechanisms

It should be noted that most of the previous studies on human aging concentrated on brain *function* or *functional* connectivity for middle-aged and/or old-aged individuals. Very few studies have focused on age-related *structural* changes in the brain. As such, there was a lack of reported matrices (i.e., cortical thickness, cortical surface area, and cortical volume) sensitive to the identification of early structural brain changes for healthy younger adults, especially in various pre-defined functional networks. Previously, in a study of 207 healthy adults between 23 and 87 years of age, Storsve and colleagues reported age related annual decrease in thickness, volume, and area in most brain areas (Storsve et al., 2014). In another study of 974 individuals (aged between 4.1 and 88.5 years), Fjell and colleagues reported that genetic factors, genetic organization and maturation of brain areas affect cortical changes throughout the life span (Fjell et al., 2015). To our knowledge, our study is the first to focus on changes in all three morphometric measures in individual functional brain networks of young healthy adults. Although the underlying mechanisms of reduction in functional and structural measures have not been explored in detail, it is suggested that the basis of this significant reduction could be due to non-pathological processes such as transition of neurons to neurofibrillary tangles and reduction in dendritic arborization or pruning (Morrison and Hof, 1997). On the other hand, it is also possible that such declines could be due to pathological processes such as amyloid deposition or unwanted chemical changes such as disorders in glucose metabolism over time (Walhovd et al., 2014). A blend of decreased neural specificity and white matter integrity could be another possible explanation of functional and structural loss with aging (Park et al., 2004). Since post mortem studies and studies on non-human primates found relatively comparable neuron count between older and younger participants, neuron death has not been suggested as a cause for reduction in magnitude of cortical measures (Morrison and Hof, 1997; Peters et al., 1998).

### Inter-dependence of CT, CSA, and CV

Consistent with a study by Lemaitre et al. (2012), we did not find a functional network with pronounced reduction in CSA with aging. This suggests that CSA is less sensitive to morphometric changes with aging, which makes CT and CV relatively more informative measures to identify age-related morphometric changes across the brain (Lemaitre et al., 2012). Lemaitre and colleagues make an analogy to a dry apple, stating “When an apple dries, its flesh and thickness reduce and its skin shrivels keeping the surface area relatively constant but gaining spatial complexity from a flat to an uneven surface” helps to better understand the possible mechanisms underlying these cortical changes (Lemaitre et al., 2012, p. 617.e7). This underlines an idea that with age, an atrophied brain might display an increase in gyral complexity without a decrease in surface area. Consistent with this hypothesis, Rettmann and colleagues also observed age-related reduction in thickness and volume but no change in surface area among eight sulcal regions (Rettmann et al., 2006). Since cortical volume, simply put, is the product of the thickness and surface area, so changes in volume account for changes in thickness as well as surface area, which could be one of the possible reasons for an inconsistent pattern of age-related changes in CV across functional networks, especially before and after considering the effect of ‘gender.’ In the field of imaging genetics, it has been suggested that methods providing measures of gray matter volume could be less sensitive for gene identification than the methods providing CT and/or CSA (Winkler et al., 2010). In addition, we did not observe significant inter-dependence between CT and CSA, reflecting the idea that the sensitivity of thickness and area to normal aging could be entirely different depending on aging and/or type of neuropsychiatric disorder (Lemaitre et al., 2012) and/or type brain structure under study. Therefore, non-uniformity in age-related changes in CT, CSA, and CV but uniformity in age-related changes in CT, dependence of CV on CT and CSA and independence of CT and CSA in the present set of functional networks for relatively younger participants reflects the importance of analyzing multiple aspects of age-related cortical changes. For instance, individual measures of CT, CSA, and CV in isolation are not enough to distinguish the age-related susceptibility of functional brain networks to completely understand the effect of normal aging on cortical measures across the whole brain. While each of these measures contributes unique and important information regarding specific age-related histological changes in brain, when used in combination, these measures provide substantially greater precision in characterizing these associations.

## Conclusion

The findings reported in this study suggest that the limbic network could be one of the most age-resistant functional networks of the human brain in young adulthood, showing no significant change in cortical characteristics, especially in CT, while other networks showed clear changes with age. This relative resistance of the primary emotional processing network could potentially serve an adaptive function to protect the individual by sustaining more primitive defensive behaviors regardless of the integrity of cortical capacities. Findings also highlight that, compared to CSA and CV, CT can be used as one of the more reliable biomarkers to identify early cortical changes in younger adults. These findings also suggest that due to the significant inter-dependence of cortical measures, it is critical to include analyses of multiple cortical measures simultaneously in order to obtain a precise and comprehensive understanding of age related changes. We speculate that neuro-markers such as the cortical measures used herein could be applied to predict possible onsets of neurodegenerative diseases in relatively older individuals, although this was not specifically explored in the present study. This study is not without limitations, however. First, the age range over a relatively small sample size in the present study is quite widespread and while there were approximately equal number of participants in each age group, this might nevertheless account for some of the observed instability across functional networks. Future studies with a focus on younger adults, involving a more narrowed age range might show increased stability of cortical measures across networks, indicating specific associations between cortical characteristics and functional networks at different ages. Second, future studies would benefit from inclusion of behavioral measures corresponding to each of the functional networks, which would further help to explore the effect of healthy aging on the relationship between age-related structural brain changes and corresponding changes in behavior and cognition. With due consideration to these limitations, we believe the present findings demonstrate important age-related associations in cortical structure even in young adulthood.

## Author Contributions

SB conducted the neuroimaging analyses and wrote the initial draft of the manuscript and organized the revisions. AA and ND each contributed to the writing of revisions of the manuscript. WK designed the study, oversaw data collection and analysis, and contributed to writing revisions of the manuscript.

## Conflict of Interest Statement

The authors declare that the research was conducted in the absence of any commercial or financial relationships that could be construed as a potential conflict of interest.

## Footnotes

1 http://www.alz.org/

2 https://www.cdc.gov/

3 https://www.cdc.gov/alcohol/fact-sheets/alcohol-use.htm

4 https://surfer.nmr.mgh.harvard.edu/fswiki
